# Recruitment of hypothalamic orexin neurons after formalin injections in adult male rats exposed to a neonatal immune challenge

**DOI:** 10.3389/fnins.2015.00065

**Published:** 2015-03-10

**Authors:** Erin J. Campbell, Stephanie M. Watters, Ihssane Zouikr, Deborah M. Hodgson, Christopher V. Dayas

**Affiliations:** ^1^Neurobiology of Addiction Laboratory, School of Biomedical Sciences and Pharmacy and the Centre for Brain and Mental Health Research, Hunter Medical Research Institute, University of NewcastleNewcastle, NSW, Australia; ^2^Laboratory of Neuroimmunology, School of Psychology, University of NewcastleNewcastle, NSW, Australia

**Keywords:** orexin, hypocretin, pain, nociception, formalin, lipopolysaccharide, early life stress

## Abstract

Exposure to early life physiological stressors, such as infection, is thought to contribute to the onset of psychopathology in adulthood. In animal models, injections of the bacterial immune challenge, lipopolysaccharide (LPS), during the neonatal period has been shown to alter both neuroendocrine function and behavioral pain responses in adulthood. Interestingly, recent evidence suggests a role for the lateral hypothalamic peptide orexin in stress and nociceptive processing. However, whether neonatal LPS exposure affects the reactivity of the orexin system to formalin-induced inflammatory pain in later life remains to be determined. Male Wistar rats (*n* = 13) were exposed to either LPS or saline (0.05 mg/kg, i.p) on postnatal days (PND) 3 and 5. On PND 80–97, all rats were exposed to a subcutaneous hindpaw injection of 2.25% formalin. Following behavioral testing, animals were perfused and brains processed for Fos-protein and orexin immunohistochemistry. Rats treated with LPS during the neonatal period exhibited decreased licking behaviors during the interphase of the formalin test, the period typically associated with the active inhibition of pain, and increased grooming responses to formalin in adulthood. Interestingly, these behavioral changes were accompanied by an increase in the percentage of Fos-positive orexin cells in the dorsomedial and perifornical hypothalamus in LPS-exposed animals. Similar increases in Fos-protein were also observed in stress and pain sensitive brain regions that receive orexinergic inputs. These findings highlight a potential role for orexin in the behavioral responses to pain and provide further evidence that early life stress can prime the circuitry responsible for these responses in adulthood.

## Introduction

Chronic pain is a debilitating condition for which effective treatments and the underlying neurobiological mechanisms responsible are yet to be fully identified (Dersh et al., 2002). Pain characteristically evokes emotional responses in individual sufferers and is often comorbid with negative affective states including anxiety and depression and sleep disturbances (Neugebauer et al., 2004; Katon et al., 2007). Interestingly, the normal maturation of the nociceptive system is dependent on the uninterrupted development of sensory inputs in early life (Fitzgerald, 2005). Exposure to adverse events during this sensitive period of development, when the nociceptive circuitry is undergoing fine-tuning, can precipitate maladaptive pain processing in later life. For example, in humans, early life physical stressors have been associated with an increased risk for developing chronic pain conditions in adulthood (Davis et al., 2005). Similarly, recent preclinical research conducted by our group has indicated that early life exposure to the immune challenge lipopolysaccharide (LPS) resulted in hyperalgesia in response to formalin, a model of inflammatory pain, in young rats (Zouikr et al., 2014b). Despite recent efforts, the neural circuitry modulating the increased response to pain following early life stress remains to be fully determined.

Importantly, the hypothalamus controls neuroendocrine stress responses and nociceptive processing (Hsieh et al., 1996; Matthews, 2002). Direct afferent and efferent relays connect hypothalamic nuclei to brain areas involved in the active modulation of pain and nociception such as the dorsal horn of the spinal cord and the periaqueductal gray (PAG; Holland and Goadsby, 2007; Todd, 2010). Additionally, subregions such as the perifornical and lateral hypothalamic areas are key brain structures coordinating behavioral and autonomic stress responses and receive significant corticolimbic input (Millan, 2002). Recently, a subpopulation of hypothalamic neurons which produce the neuropeptides orexins (hypocretins), have been identified as possible substrates in the modulation of pain and stress through projections to the PAG, brainstem and paraventricular thalamus (PVT; Peyron et al., 1998; Marcus et al., 2001). For example, injections of orexin-A into the rostral ventromedial medulla produced antinociceptive-like behavior in response to formalin in male rats (Azhdari-Zarmehri et al., 2014). Interestingly, maternal separation was found to alter orexin system reactivity to psychological stress in adulthood (James et al., 2014). Despite these results, only a few studies have assessed orexin system recruitment and also studied behavioral responses following noxious stimuli. Watanabe et al. (2005) demonstrated that prepro-orexin knockout mice exhibited increased nociceptive behaviors in response to peripheral inflammation and reduced stress-induced analgesia following footshock stress in adulthood compared to wild type mice. Further, Heidari-Oranjaghi et al. (2012) found that intracerebroventricular (i.c.v) injections of the OX_1_ orexin receptor antagonist SB-334867, resulted in increased pain responses to formalin, but only following both restraint and swim stress. Recently, we demonstrated that rats exposed to neonatal LPS displayed enhanced formalin-induced flinching but not licking responses in adolescence at postnatal day (PND) 22 (Zouikr et al., 2014b). These behavioral changes were accompanied by attenuated Fos-protein cell counts in the rostral dorsal PAG as well as the rostral and caudal axes of the ventrolateral PAG. But, whether exposure to early life immune challenge alters orexin system function in response to a noxious stimulus in adulthood (PND 80–97) has not been determined. This information will improve our understanding as to how neonatal physical and emotional insults can rewire the brain pathways involved in pain and stress processing.

Therefore, the aim of the present study was to assess the effect of neonatal LPS exposure on the reactivity of the orexin system, as assessed by Fos-protein expression, to formalin challenge in adulthood (PND 80–97). Given the relationship between stress, nociception and the orexin system, it was hypothesized that rats exposed to LPS in early life would demonstrate increased pain and grooming responses to formalin in adulthood. We predicted that these behavioral responses would be accompanied by evidence of increased orexin cell activity, and that this recruitment pattern would also be reflected in Fos-responses in downstream projection targets of this system.

## Methods and materials

### Ethics statement

All experiments performed were approved by the University of Newcastle Animal Care and Ethics Committee, and carried out in accordance with the National Health and Medical Research Council Australian Code of Practice for the care and use of animals for scientific practice.

### Animals

Four experimentally naïve female Wistar rats were obtained from the University of Newcastle Animal house and bred with two experimentally naïve males in the University of Newcastle vivarium. On PND 3 and 5 a random subset of animals from each litter were administered LPS as a neonatal immune challenge (detailed below). A total of 13 male offspring were included in this study, 6 LPS-exposed animals and 7 saline animals. On PND 21, animals were weaned with 2 animals/cage (41.5 × 28 × 22 cm cages; Mascot Wire Works, Sydney). Food (Rat and Mouse Pellets, Glen Forest, Western Australia) and water were available *ad libitum* and rats were maintained on a 12 h light (0600–1800): 12 h dark cycle. Temperature was maintained at 20 ± 2°C and humidity was kept at 34 ± 2%.

### Neonatal LPS challenge

The neonatal LPS procedure was performed as per previously published procedures (Walker et al., 2009). Between 0900 and 1000 h on PND 3 and 5 (birth as PND 1), pups in the LPS treatment condition were briefly removed from their home cages and administered 0.05 mg/kg LPS (intraperitoneally, i.p, LPS from *Salmonella enterica*, serotype *enteritidis*, Sigma-Aldrich, USA, dissolved in 20 μl sterile pyrogen-free saline). Saline controls were given an equal volume of sterile saline (Livingstone International, Australia). The timing of injection and dose were selected as it had been previously shown to produce a sustained immune response with no mortality (Walker et al., 2006).

### Formalin test

This test has been previously described in our laboratory (Zouikr et al., 2014c) and is a well-validated model of behavioral responses to nociceptive stimuli (early phase or phase 1, first 5 min), inhibition of nociceptive responding (interphase, 5–15 min) and inflammation (late phase or phase 2, 15–60 min; Wheeler-Aceto and Cowan, 1991; Tjølsen et al., 1992; Henry et al., 1999; Fischer et al., 2014). Between PND 80 and 97 animals were removed from their home cage and a subcutaneous injection of formalin (2.25%) was administered into the plantar surface of the right hindpaw of all rats (50 μl formaldehyde 36.5–38%; Biolab Ltd, Victoria, Australia; sodium chloride injection BP 0.9% Pfizer, Australia). This volume and concentration of solution has been previously shown by our group to produce the biphasic response of the formalin test (Zouikr et al., 2013). A saline control injection into the hindpaw was not included, as this has been found to produce no pain-induced behaviors including licking and flinching (Guy and Abbott, 1992; Butkevich and Vershinina, 2001). The behavioral response to formalin was examined in transparent plexiglas boxes (30 × 30 × 30 cm) for 1 h. A researcher blind to experimental conditions scored behavioral responses using JWatcher ethograph software (version 0.9, Macquarie University, Sydney, Australia). Pain behaviors were measured by the number of flinches of the injected paw and the time spent licking the injected paw. Exploratory behavior was measured as the time spent rearing during the formalin test and grooming behavior during the formalin test was analyzed as the time spent grooming the forepaws.

### Immunohistochemistry

Ninety minutes following formalin injections, rats were deeply anaesthetized with an overdose of sodium pentobarbitone (200 mg/kg; i.p; Virbac, Australia) and transcardially perfused with 200 mL of 0.1 M phosphate buffered saline followed by 500 mL of 4% paraformaldehyde (pH 9.5). Brains were then removed and postfixed in 4% paraformaldehyde (24 h at 4°C) and then stored in 15% sucrose until sectioning. Serial coronal sections of the rostral forebrain (40-μm) and caudal midbrain (50-μm) were cut using a freezing microtome (Leica Microsystems, SM2000R). A 1-in-4 series of sections from the hypothalamus (bregma −2.28 to −3.24), the PVT (bregma −2.28 to −3.24), the paraventricular nucleus of the hypothalamus (PVN; bregma −1.46 to −1.78) and the amygdala (bregma −2.28 to −3.08), and a 1-in-5 series of sections from the PAG (bregma −6.69 to −8.19) were processed for immunohistochemical detection of Fos-protein (72 h, 1:10000, rabbit polyclonal, sc-52, Santa Cruz Biotechnology, CA, USA) as described previously in detail (Dayas et al., 2008; James et al., 2014). Following primary antibody application, sections were incubated in a secondary antibody (2 h, 1:300, donkey anti rabbit, 711-065-152, Jackson IR, PA, USA). Hypothalamic sections were dual-labeled for orexin-A, also likely detecting pre-pro orexin (48 h, 1:15000, orexin-A antibody, goat polyclonal, sc-8070, Santa Cruz Biotechnology). The selectivity of this antibody has been illustrated in a recent study by Blanco-Centurion et al. (2013). Please see Supplementary Material S1 outlining the details of the specificity of the orexin-A antibody. Following orexin primary antibody application, sections were subsequently incubated in a secondary antibody (2 h, 1:400, donkey anti goat, 705-065-147, Jackson IR, PA, USA). An equal number of animals from each treatment group were included in each individual immunohistochemical run.

Bilateral counts of single-labeled Fos-positive cells were made in the PVT, PVN, basolateral amygdala (BLA), medial nucleus of the amygdala (MeA), the central nucleus of the amygdala including both lateral and medial subdivisions (CeL, CeM respectively), the dorsal PAG (including both the dorsomedial and dorsolateral columns), and the lateral and ventrolateral PAG using Metamorph Imaging System Software (Version 7.5; Molecular Devices Analytical Technologies) at 10x magnification (Olympus CX40). Quantification of Fos-positive cells was determined by creating a region of interest for each brain structure and a thresholding procedure was used to quantify Fos expression. Counts of Fos-positive orexin cells were made in the dorsomedial hypothalamus (DMH), the perifornical area (PFA), and the lateral hypothalamus (LH) by one observer, blind to treatment, using a 20x microscopic objective (Olympus CX40). The DMH was defined as the area between the third ventricle and the medial side of the PFA, the PFA was defined as the area surrounding the fornix and the LH was defined as the area from the lateral side of the PFA to the optic tract (Laorden et al., 2012; James et al., 2014). All brain coordinates were adapted from the Paxinos and Watson atlas (Paxinos and Watson, 2007).

### Data analysis

Initial analysis of covariance (ANCOVA) analyses revealed no significant effect of litter size on both behavioral and brain comparisons. Behavioral data was analyzed across neonatal treatment group using one-way between subjects ANOVAs for each phase of the formalin test. Using area under the curve calculations for the formalin test, phase 1 was considered the first 5 min, the interphase was the sum of 6–15 min, and phase 2 was the sum of responses from 16–60 min. Fos-protein immunohistochemical data was analyzed using two-way between subjects ANOVAs comparing neonatal treatment and brain region where appropriate, alternatively one-way ANOVAs were used. *Post-hoc* comparisons were assessed using least significant differences tests. Pearson's correlations were used to examine the relationship between the percentage of Fos-positive orexin cells in the subregions of the hypothalamus and behavioral responses of animals in phase 1 and the interphase of the formalin test. All statistical analyses were conducted using IBM SPSS V21 with an alpha value of 0.05. All figures are represented as means with standard errors.

## Results

### Effect of neonatal LPS exposure on formalin-induced nociceptive behavior

**Figure 1:**
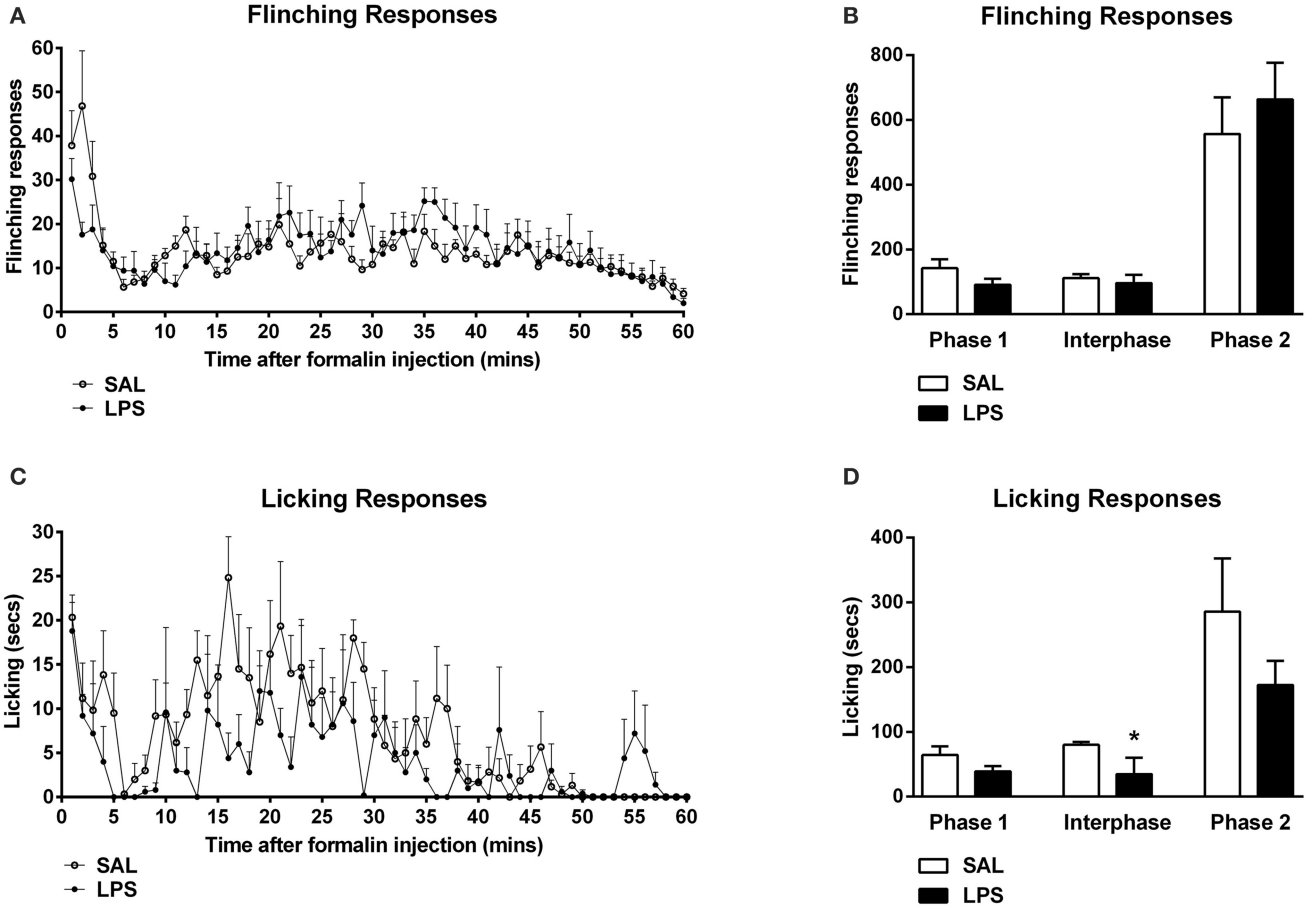
**Neonatal LPS exposure enhanced pain suppression of licking behaviors during the interphase in response to formalin in adulthood**. Time course of flinching and licking responses for LPS and saline rats exposed to formalin in adulthood **(A,C)**. No differences were observed in flinching behavior across any phase of the formalin test between LPS-treated rats and saline controls **(B)**. LPS-exposed rats exhibited a potentiated inhibitory pain response to formalin in licking behaviors during the interphase, with no effect of neonatal treatment on phase 1 or phase 2 of the formalin test **(D)**. Data are presented as mean + standard error. ^*^p < 0.05. SAL: *n* = 7; LPS: *n* = 6.

One-way between subjects ANOVAs revealed no significant effect of neonatal treatment on flinching behavior in any phase of the formalin test (*p*'s > 0.05; Figures 1A,B). An analysis of licking responses revealed a significant effect of neonatal treatment during the interphase with LPS animals displaying reduced time spent licking compared to saline controls [F_(1, 12)_ = 3.795, *p* = 0.042; Figures 1C,D].

### Effect of LPS on formalin-induced exploratory and grooming behaviors

**Figure 2:**
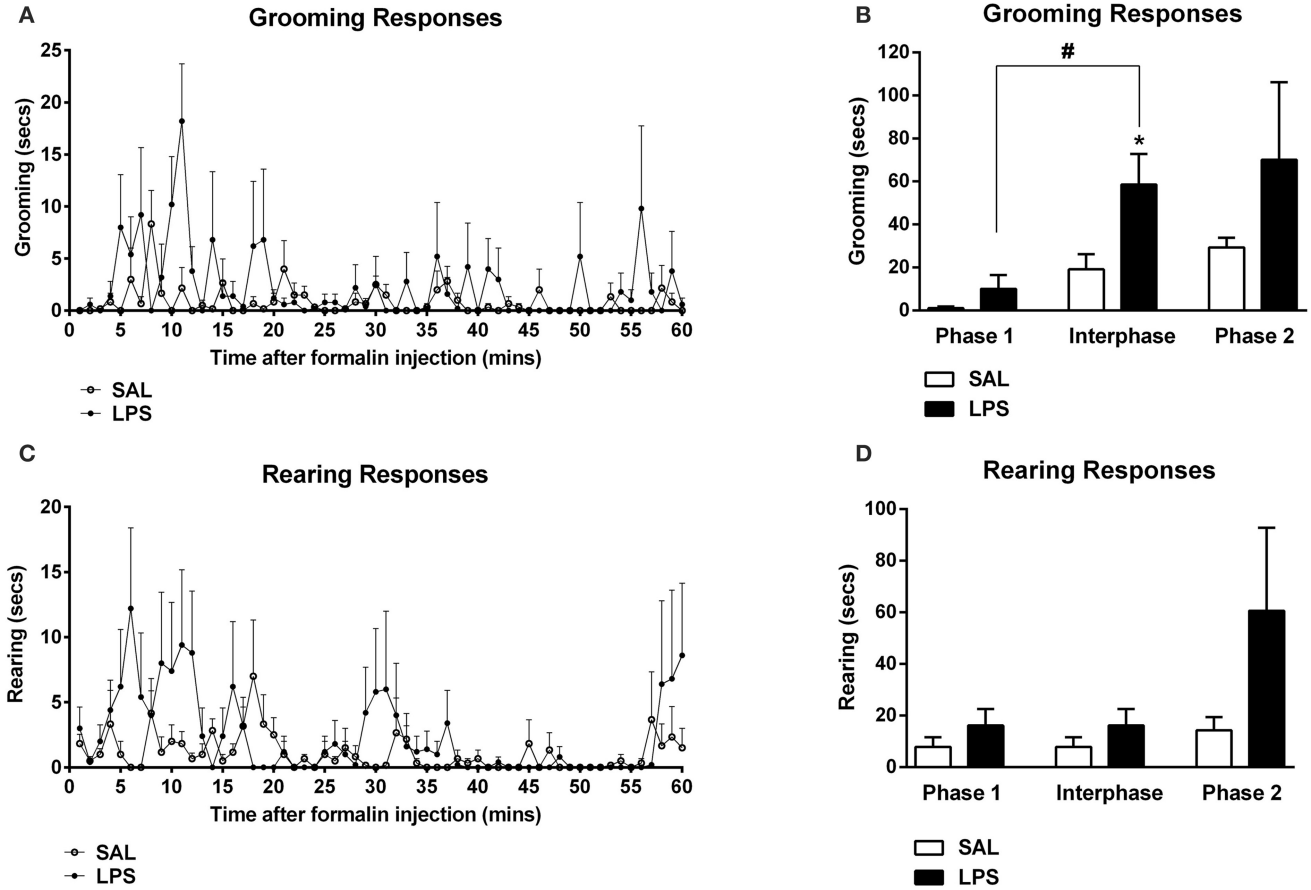
**An early life immune challenge increased self-grooming responses to formalin in adulthood**. Time course of grooming and rearing responses for LPS and saline animals **(A,C)**. LPS-treated rats displayed increased grooming behaviors in response to formalin when compared to saline animals **(B)**. There was no effect of neonatal treatment on rearing behavior across all phases of the formalin test **(D)**. Data are presented as mean + standard error. ^*^p < 0.05, #*p* < 0.05 phase 1 vs. interphase. SAL: *n* = 7; LPS: *n* = 6.

One-way between subjects ANOVAs revealed a significant effect of neonatal treatment on the total time spent grooming in the interphase of the formalin test with LPS-treated animals spending more time grooming compared to saline animals [F_(1, 12)_ = 6.96, *p* = 0.014; Figures 2A,B]. LPS-treated animals also displayed significantly increased time grooming from phase 1 to the interphase of the formalin test compared to saline animals [F_(1, 12)_ = 5.538, *p* = 0.022; Figure 2B]. ANOVA also revealed no significant effects of neonatal treatment on time spent grooming during phase 1 or phase 2, nor for time spent rearing in any phase of the formalin test (*p*'s > 0.05; Figures 2C,D).

### Effect of neonatal immune challenge on orexin cell reactivity in response to formalin in adulthood

**Figure 3:**
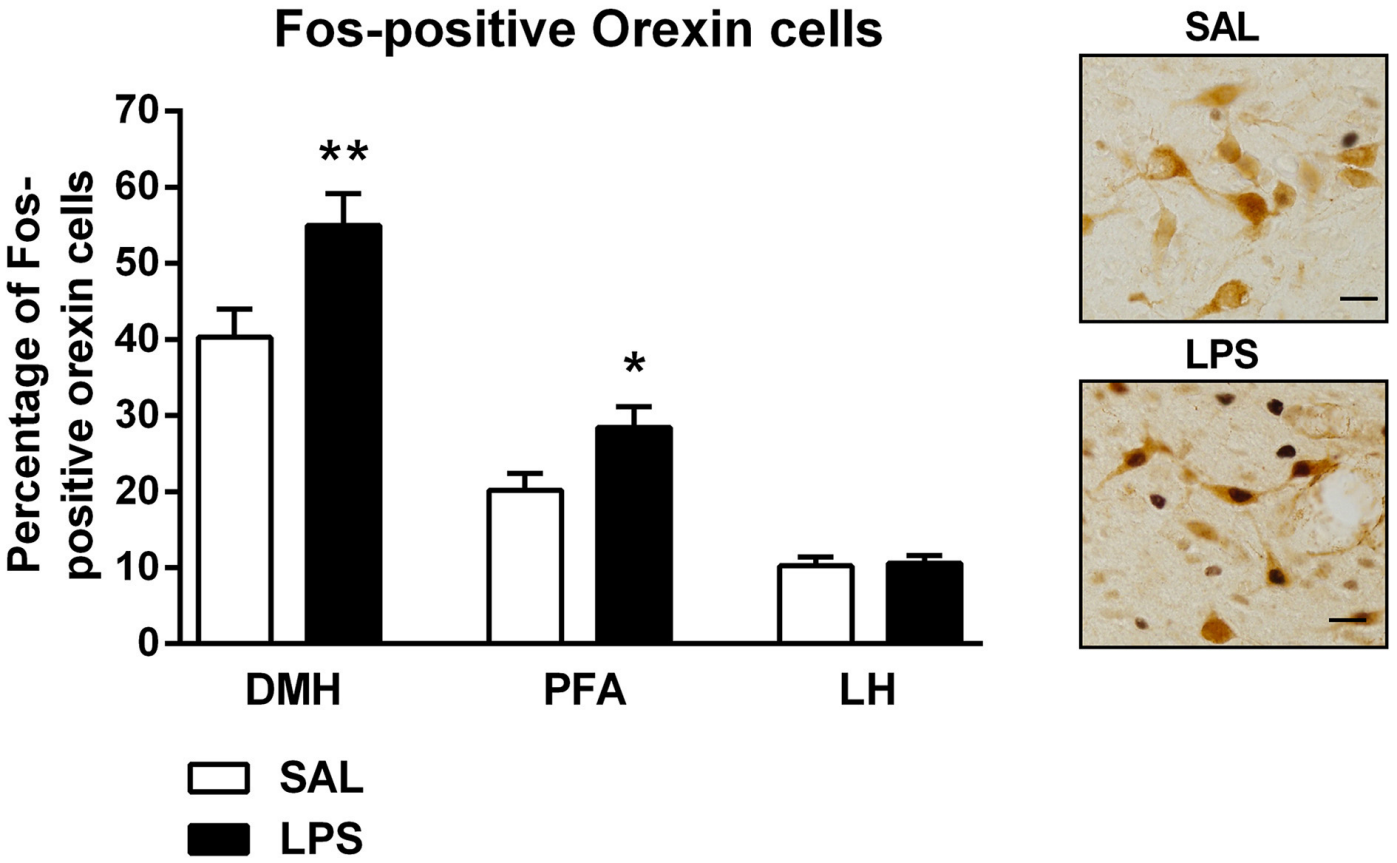
**LPS resulted in an increase in the percentage of Fos-positive orexin cells in both the dorsomedial (DMH) and perifornical (PFA) subregions of the hypothalamus**. The percentage of Fos-positive orexin cells in the DMH and PFA was significantly higher in LPS-exposed animals compared to saline controls. No differences were observed in orexin cell activity in the lateral hypothalamus (LH) across neonatal treatment groups. Photomicrographs of coronal sections of the hypothalamus immunolabeled for Fos-protein and orexin. Data are presented as mean + standard error. ^*^p < 0.05, ^**^p < 0.01, scale bar 20 μm. SAL: *n* = 6; LPS: *n* = 6.

There were no differences found in the total number of orexin-positive cells between LPS animals and saline controls in any subregion of the hypothalamus (*p*'s > 0.05, Table 1). However, a two-way between subjects ANOVA revealed a significant main effect of neonatal treatment on the percentage of Fos-positive orexin cells with LPS-exposed rats exhibiting a greater percentage of Fos-positive orexin cells compared to saline-treated animals [F_(1, 246)_ = 11.863, *p* = 0.001, Figure 3]. Additionally, there was a significant interaction between neonatal treatment and hypothalamic subregion on the percentage of Fos-positive orexin cells [F_(2, 246)_ = 3.387, *p* = 0.035]. *Post-hoc* comparisons revealed that LPS animals displayed significantly greater percentages of Fos-positive orexin cells in the DMH and PFA compared to saline controls (*p*'s < 0.05) however, no differences were found in the LH (*p* > 0.05, Figure 3).

**Table 1:** **Orexin cell expression in each subregion of the hypothalamus**.

**Treatment**	**Region**	**Orexin number**	**Fos-Orexin number**
Saline (*n* = 6)	DMH	30.77±3.65	11.97±1.55
	PFA	111.33±10.56	19.99±2.71
	LH	35.18±2.33	3.35±0.41
LPS (*n* = 6)	DMH	30.89±2.85	16.89±2.34
	PFA	101.59±7.02	29.62±3.77
	LH	39.60±3.13	4.72±0.63

*Data presented as average number of orexin cells or Fos-positive orexin cells ± standard error mean in the dorsomedial hypothalamus (DMH), perifornical area (PFA), and lateral hypothalamus (LH)*.

### Effect of LPS on Fos-protein expression in the PVT, PVN, amygdala and PAG

**Figure 4:**
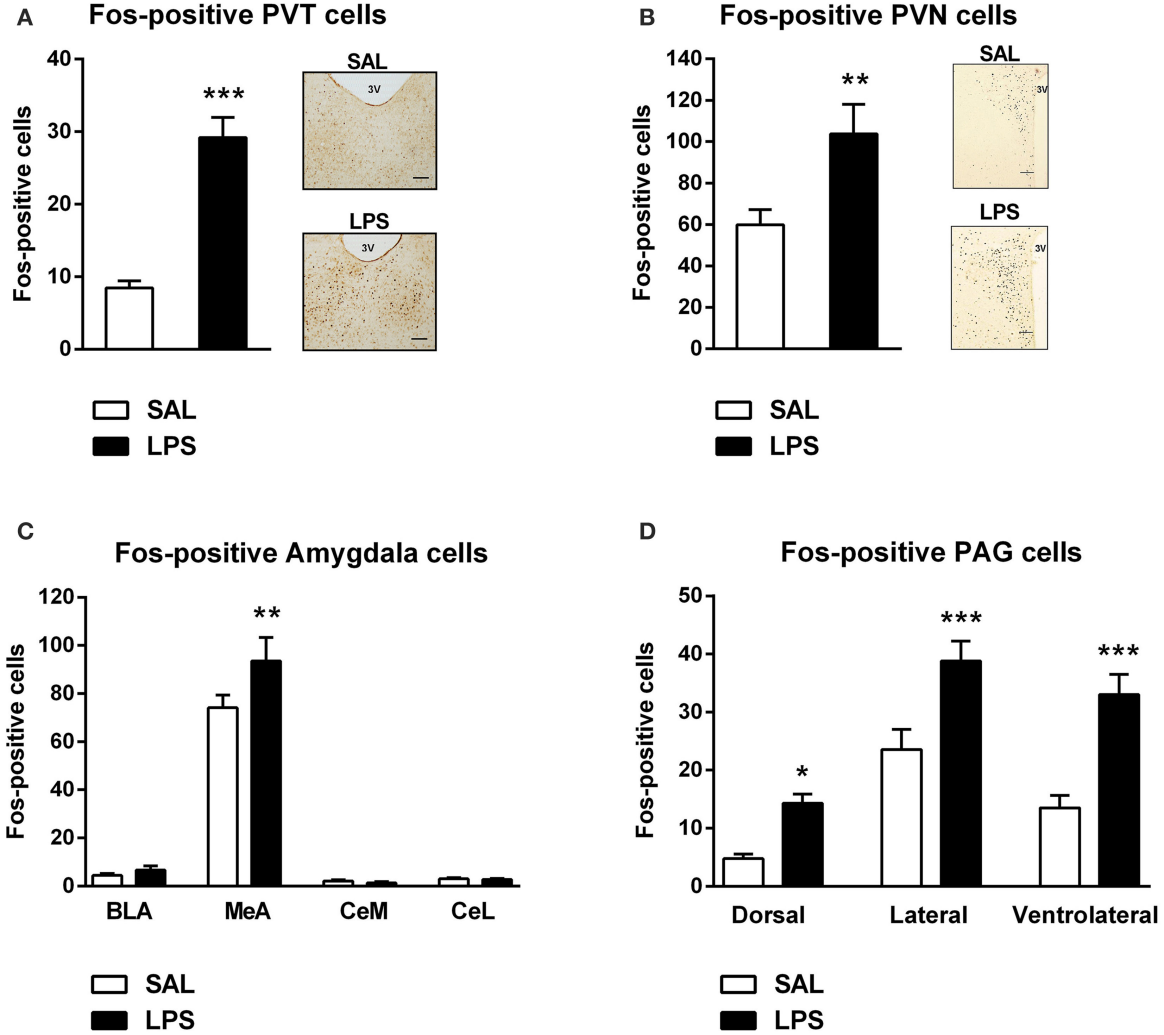
**Neonatal LPS treatment resulted in an increase in the number of Fos-positive cells in the paraventricular thalamus (PVT), paraventricular nucleus (PVN), the medial nucleus of the amygdala (MeA) and the periaqueductal gray (PAG)**. Early life LPS treatment resulted in an increase in Fos activity in both the PVT and PVN **(A,B)**. Photomicrographs of coronal sections of the PVT and PVN immunolabeled for Fos-protein **(A,B)**. LPS-treated animals also exhibited an increased number of Fos-positive cells in the MeA **(C)**. There was no effect of neonatal treatment on any other subregion of the amygdala. LPS-exposed rats also had increased Fos-protein expression in the dorsal, lateral and ventrolateral PAG **(D)**. Data are presented as mean + standard error. ^*^p < 0.05, ^**^p < 0.01, ^***^p < 0.001, scale bar 100 μm, 3V = third ventricle. SAL: *n* = 6; LPS: *n* = 6.

A one-way between subjects ANOVA revealed that LPS-treated animals exhibited significantly greater numbers of Fos-positive cells in the PVT and PVN compared to saline controls [F_(1, 82)_ = 59.055, *p* <0.001; F_(1, 31)_ = 9.370, *p* = 0.005; Figures 4A,B].

A two-way between subjects ANOVA revealed a significant interaction of neonatal treatment and amygdala subregion on the number of Fos-positive cells [F_(3, 280)_ = 2.896, *p* = 0.036]. Least significant differences comparisons revealed that LPS-treated animals displayed a significantly greater number of Fos-positive cells in the MeA compared to saline animals (*p* = 0.001; Figure 4C).

A two-way between subjects ANOVA revealed a main effect of neonatal treatment on the number of Fos-positive cells within the PAG [F_(1, 204)_ = 38.440, *p* < 0.001]. *Post-hoc* comparisons revealed that LPS-treated animals displayed significantly greater numbers of Fos-positive cells in the dorsal (*p* = 0.022), lateral (*p* < 0.001) and ventrolateral PAG (*p* < 0.001) compared to saline animals (Figure 4D).

### Correlations between orexin cell activity and behavioral responses to formalin in adulthood

**Figure 5:**
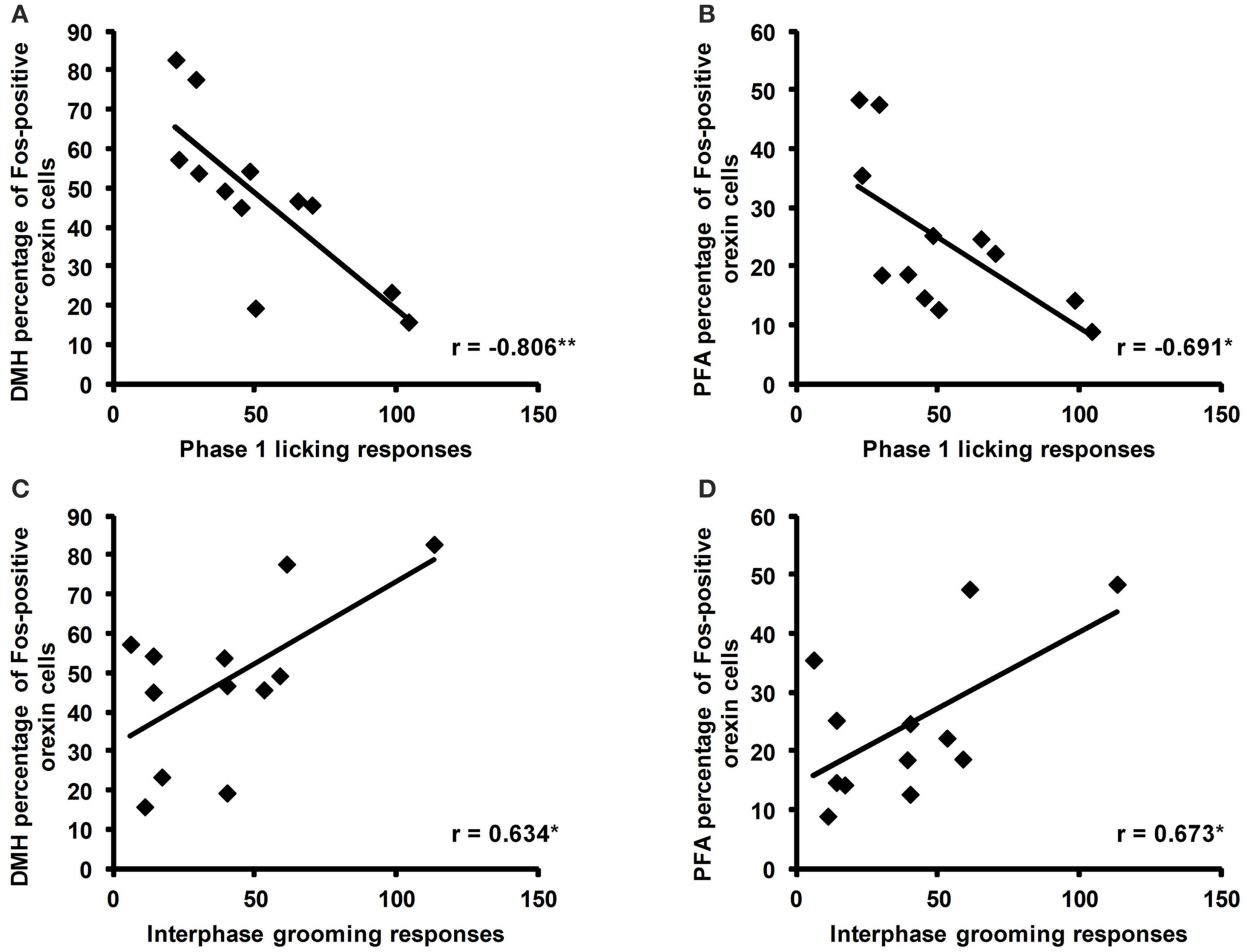
**Correlations of the percentage of Fos-positive orexin cells with licking and grooming behaviors during the formalin test**. Significant negative correlations were found between the time spent licking (secs) during phase 1 of the formalin test and the percentage of Fos-positive cells in the dorsomedial hypothalamus (DMH) and perifornical area (PFA; **A,B**). Significant positive correlations were found between the time spent grooming (secs) during the interphase of the formalin test and the percentage of Fos-positive orexin cells in the DMH and PFA **(C,D)**. ^*^p < 0.05, ^**^p < 0.01. SAL: *n* = 6, LPS: *n* = 6.

Correlation analyses revealed a negative correlation between the total time spent licking in phase 1 of the formalin test and the percentage of Fos-positive orexin cells in the DMH (*r* = −0.806, *p* = 0.005) and the PFA (*r* = −0.691, *p* = 0.027; Figures 5A,B). In the interphase of the formalin test, total time spent grooming was also positively correlated with the percentage of Fos-positive orexin cells in the DMH (*r* = 0.634, *p* = 0.049) and PFA (*r* = 0.673, *p* = 0.033, Figures 5C,D).

## Discussion

In the current study we show that animals exposed to LPS in early life exhibit altered behavioral responses to a formalin challenge in adulthood. LPS-treated animals displayed increased orexin cell activity, as assessed by Fos-like immunoreactivity in the DMH and PFA but not LH. Additionally, increases in numbers of Fos-positive neurons were observed in stress and pain sensitive brain regions that express orexin receptors including the PVT, PVN, MeA, and PAG.

### Increased orexin cell reactivity to formalin following an early life immune challenge

The primary aim of this study was to examine the response of orexin neurons to an acute formalin injection in adulthood following early life LPS exposure. Using Fos-protein immunohistochemistry, we observed an increase in the recruitment of DMH and PFA orexin neurons in LPS-exposed rats compared to controls. Interestingly, no change in orexin cell reactivity to formalin was observed in the LH. This differential recruitment pattern is interesting given the recent suggestions of a dichotomy of function between medial and lateral orexin cell populations (Estabrooke et al., 2001; Harris and Aston-Jones, 2006). For example, orexin neurons in the DMH and PFA have been linked with arousal and the modulation of the stress response whereas those located in the LH have been linked with reward (Harris et al., 2005). In support, rats administered sodium-lactate to induce panic anxiety exhibited increased activation of DMH-PFA but not LH orexin neurons (Johnson et al., 2010). Thus, arousal and stress responsive orexin cells may be preferentially sensitized by early life immune stress.

It is unclear from the present study which pain sensitive afferent pathways may have been involved in the recruitment of orexin neurons. Brain sites that receive direct afferent inputs from lamina I projection neurons in the superficial dorsal horn include the ventrolateral medulla, nucleus of the solitary tract, lateral parabrachial nucleus, PAG and the thalamus (Millan, 2002; Gauriau and Bernard, 2004; Todd, 2010). The parabrachial nucleus is interesting in this regard possessing efferent projections to the amygdala and the LH (Bernard et al., 1993; Bester et al., 1997; Gauriau and Bernard, 2002). These inputs may directly recruit orexin neurons. Additionally, the spinal cord sends direct projections to the hypothalamus. For example, Burstein et al. (1987) demonstrated direct projections from three regions of the spinal gray matter, including the lateral reticulated area, the area surrounding the central canal and the marginal zone, to the hypothalamus (Burstein et al., 1987; Giesler, 1995). However, the direct input from these spinal cord regions to orexin neurons is yet to be examined. Interestingly, the stress responsive central nucleus of the amygdala (CeA) and MeA also project to the LH (Peyron et al., 1998; Yoshida et al., 2006) and may provide “top-down” afferent input to the LH. Clearly, further work will be required to determine whether the activation of orexin neurons occurs through ascending nociceptive pathways or descending inputs from stress responsive centers such as the amygdala. It is important to acknowledge that because previous work has shown that hindpaw saline injections cause no pain-induced behaviors including licking and flinching (Guy and Abbott, 1992; Butkevich and Vershinina, 2001), we did not include a Fos control group for the formalin challenge. While it is difficult to determine the direction of change from baseline in our Fos-induced orexin cell reactivity, prior studies have shown that rats subjected to hindpaw saline injections showed no Fos labeling in the lumbar spinal cord (Yi and Barr, 1995). Additionally, Barr (2011) reported that 14 day old rat pups given saline into their hindpaw did not exhibit Fos labeling in the PVN or dorsal/lateral PAG.

### Enhanced formalin-induced inhibitory pain response in LPS-treated rats

The formalin test is a well-established animal model of persistent pain (Tjølsen et al., 1992). Three distinct behavioral responses are commonly associated with the formalin test. The early phase, or phase 1, involves the direct chemical stimulation of nociceptors, the interphase denotes the active inhibition of pain and the late phase, or phase 2, represents the inflammatory pain response (Dubuisson and Dennis, 1977; Tjølsen et al., 1992; Franklin and Abbott, 1993; Henry et al., 1999; Fischer et al., 2014). Importantly, of these three phases, the interphase has received the least attention. Here, we demonstrated decreased licking behaviors in the interphase after formalin challenge in LPS animals. This is perhaps not surprising given that increased grooming may override or mask changes in licking behavior. Although a slight trend was observed in phase 2 we found no significant changes in flinching behavior in response to formalin between treatment groups in male rats. This is in contrast with our recently published work whereby adult rats exposed to a neonatal LPS challenge displayed a significantly increased flinching response during the late phase of the formalin test (Zouikr et al., 2014a). This discrepancy could be attributed to methodological differences, presently, we analyzed the interphase whereas Zouikr et al. (2014a) focused on phase 2 of the formalin test. Further, differences in behavioral scoring methods (manual vs. software) may have contributed to this discrepancy. Lastly, enhanced grooming in the current cohort may have masked changes in flinching. The putative enhancement of pain suppression observed in the interphase of LPS-treated rats is interesting given the increase in orexin cell reactivity in LPS-exposed rats. Furthermore, overall licking behaviors of LPS-treated rats negatively correlated with the percentage of Fos-positive orexin cells in the DMH and PFA. These findings are consistent with data implicating the orexin system in descending inhibitory pain pathway control (Bingham et al., 2001).

In the current study we also observed an increase in Fos-protein in all subregions of the PAG in neonatally LPS-treated rats compared to controls. It is important to note that the PAG is anatomically organized into longitudinal columns including the dorsal PAG, the lateral PAG and the ventrolateral PAG and each column plays distinct roles in the response to both stress and pain (Bandler and Shipley, 1994). The PAG has a well-characterized role in analgesia and stress coping and is the recipient of orexinergic innervation (Peyron et al., 1998; Keay and Bandler, 2002; Gebhart, 2004; Chapman et al., 2008). It is possible that the increase in orexin cell activity and the enhanced inhibitory pain response to formalin contribute to the active inhibition of pain through projections to the PAG. In support, Azhdari-Zarmehri et al. (2011) found that microinjections of orexin-A into the PAG enhanced inhibitory pain responses in the interphase in response to formalin in adulthood. Indeed, orexin-A has been shown to reduce inhibitory postsynaptic currents in ventrolateral PAG neurons that directly project to the rostral ventromedulla (Ho et al., 2011). Of the PAG columns, the lateral PAG tends to receive stronger orexin inputs, which is interesting given its role in active coping strategies in the response to pain (Bandler and Shipley, 1994; Yoshida et al., 2006). Accordingly, the pattern of Fos activity we observed in the PAG may reflect affective coping strategies in response to formalin-evoked stress. For example, Keay and Bandler (2001) demonstrated that increased Fos activity in both the ventrolateral and lateral PAG is linked with altered emotional coping responses to persistent pain. These results may help explain the affective behavioral changes observed in response to formalin as described in more detail below.

### Early life LPS evoked an affective-like behavioral response to formalin

We also examined the affective-like responses to formalin in adulthood by assessing both grooming and rearing. Self-grooming is thought to reflect a coping mechanism to produce de-arousal (Spruijt et al., 1992; Kalueff and Tuohimaa, 2005; Lariviere et al., 2011). In our study, LPS-exposed animals spent significantly more time grooming in response to formalin in adulthood compared to saline controls. No differences were observed in rearing behavior. In keeping with our findings, Aloisi et al. (1998) demonstrated that exposure to acute restraint stress in adulthood increased self-grooming during the interphase of the formalin test. Interestingly, our study found that an increase in orexinergic activity was correlated with an increase in grooming behavior following formalin injection. These data are in line with previous research implicating dysregulated orexin system function in affective behavioral responses to stress or adverse experiences in adulthood (Johnson et al., 2010; Li et al., 2010; James et al., 2014; Yeoh et al., 2014). Further, Low and Fitzgerald (2012) have demonstrated an increase in the number of Fos-positive orexin cells in animals exposed to neonatal pain followed by later life pain. Low and Fitzgerald (2012) also found this orexinergic activity to be negatively correlated with rearing behavior. Together these results suggest that the orexin system may be susceptible to early life immune or emotional challenges, which promote neuroadaptations that manifest as dysregulated behavioral and neural responses to painful stimuli in later life.

### Fos-protein expression in stress responsive brain regions

We identified an increased pattern of Fos-protein in brain regions that are known to receive strong orexinergic input and are involved in the neuroendocrine and behavioral response to stress or pain modulation (Peyron et al., 1998; Marcus et al., 2001; Vanegas and Schaible, 2004). The brain regions we examined were the PVT, PVN, amygdala and PAG. LPS-exposed animals displayed increased Fos-protein expression in the PVT and PVN. The PVN and PVT both play an important role in the neuroendocrine and autonomic responses to stress. PVN corticotrophin-releasing factor cells constitute the apex of the hypothalamic-pituitary-adrenal (HPA) axis and the PVT is involved in regulating the HPA axis response to chronic stressors (Bhatnagar and Dallman, 1998; Dayas et al., 2004; Kirouac et al., 2005). Increased Fos immunoreactivity in the PVN and PVT is therefore consistent with other studies demonstrating activation of these stress response systems to a variety of physical stressors including painful stimuli such as cold and formalin-induced pain (Pacák and Palkovits, 2001).

Surprisingly, we found no changes in the numbers of Fos-positive CeA nuclei between treatment groups. This result contrasts previous research demonstrating increased CeA activity in response to physical stressors including persistent pain and acute pain or stress (Dayas et al., 2001; Neugebauer et al., 2004). Notably, orexin-immunoreactive fibers and orexin receptors are also observed in other subregions of the amygdala including the MeA (Peyron et al., 1998; Marcus et al., 2001). The MeA is typically sensitive to psychological stressors and has recently been identified as a central site mediating repetitive self-grooming behaviors, which has linked it to a range of neuropsychiatric disorders (Dayas et al., 1999, 2001; LeDoux, 2000; Hong et al., 2014). Interestingly, we observed a significant increase in Fos-positive nuclei in the MeA of LPS-exposed animals, an effect associated with elevated self-grooming responses to formalin. It is possible that ascending spinoparabrachial projections may recruit an orexin → MeA pathway resulting in affective-like behavioral responses to formalin. However, the MeA also provides input to DMH/PFA and may be recruited by ascending pain sensitive-pathways. Further work will be required to understand the hierarchical sequence for how these brain regions are recruited by formalin.

Taken together, the behavioral data presented here confirmed that animals exposed to an early life immune challenge exhibited an enhanced inhibition of pain during the interphase. These changes were associated with increased grooming behavior, which was strongly correlated with numbers of Fos-positive orexin neurons in the DMH/PFA. Our results are interesting given evidence that patients suffering from chronic pain disorders tend to suffer more from the affective disturbances of pain than frank pain itself (Crombez et al., 1999). Further, processes modulated by the orexin system eg. sleep, feeding, and motivation, are often disturbed in people suffering chronic pain states (Dersh et al., 2002). Thus, pharmacological or non-pharmacological interventions that restore normal orexin system function may prove beneficial in the treatment of chronic pain states.

### Conflict of interest statement

The authors declare that the research was conducted in the absence of any commercial or financial relationships that could be construed as a potential conflict of interest.
