# Supplementary material for: Recruitment of hypothalamic orexin neurons after formalin injections in adult male rats exposed to a neonatal immune challenge
Source: Front Neurosci. 2015 Mar 10;9:65. doi: 10.3389/fnins.2015.00065 (PMC4354278; doi:10.3389/fnins.2015.00065)
Supplement: Supplementary file 1 [file Presentation1.PDF]

Supplementary Material – S1

Immunohistochemistry performed with the Santa Cruz orexin A goat polyclonal antibody produced amber cytoplasmic cellular profiles that were restricted to rostrocaudal levels -2.28 to -3.24, the known rostro-caudal distribution of orexin neurons (Cutler et al., 1999). Further, the pattern of labelling we observed was entirely consistent with the original description of orexin-immunoreactive cells in the lateral hypothalamus by Peyron et al., 1998 and, immunohistochemical detection of putative orexin neurons performed in our laboratory using a different antibody - a rabbit polyclonal antibody orexin A (Rb) from Phoenix Pharmaceuticals H-003-30 (Phoenix). To validate the selectivity of our immunohistochemistry for orexin, multiple controls were used, including a close examination of sections co-labelled for CART/MCH and orexin which detected intermingled but non-overlapping populations of orexin-positive cells restricted to the lateral hypothalamus, please see Dayas et al., (2008), Yeoh et al., (2012), and James et al., (2014). Importantly, a recent study Blanco-Centurion et al., 2013 showed that in pre-pro orexin knock-out mice orexin A immunoreactive terminals and neuronal profiles were completely absent after processing tissue for immunohistochemistry using the same Santa Cruz orexin antibody used in our study (personal communication). It should also be noted that sections used for analysis of Fos-only counts in the hypothalamic PVN and thalamic PVT were also immunolabelled for orexin, please see Figure 4. Consistent with above - only terminals and no cell bodies were detected in these regions.

References

Blanco-Centurion, C., Liu, M., Konadhode, R., Pelluru, D., & Shiromani, P. J. (2013). Effects of orexin gene transfer in the dorsolateral pons in orexin knockout mice. *Sleep, 36*(1), 31-40.

Cutler, D. J., Morris, R., Sheridhar, V., Wattam, T. A. K., Holmes, S., Patel, S., et al. (1999). Differential distribution of orexin-A and orexin-B immunoreactivity in the rat brain and spinal cord. *Peptides. 20,* 1455-1470.

Dayas, C.V., Mcgranahan, T. M., Martin-Fardon, R., & Weiss, F. (2008). Stimuli linked to ethanol availability activate hypothalamic CART and orexin neurons in a reinstatement model of relapse. *Biol. Psych. 63*, 152–157.

James, M. H., Campbell, E. J., Walker, F. R., Smith, D. W., Richardson, H. N., Hodgson, D. M., & Dayas, C. V. (2014). Exercise reverses the effects of early life stress on orexin cell reactivity in male but not female rats. *Front. Behav. Neurosci. 8*, 244.

Peyron, C., Tighe, D. K., van den Pol, A. N., de Lecea, L., Heller, H. C., Sutcliffe, J. G., & Kilduff, T. S. (1998). Neurons containing hypocretin (orexin) project to multiple neuronal systems. *Journal of Neuroscience, 18*(23), 9996-10015.

Yeoh, J. W., James, M. H., Jobling, P., Bains, J. S., Graham, B. A., & Dayas, C. V. (2012). Cocaine potentiates excitatory drive in the perifornical/lateral hypothalamus. *J Physiol, 590*(Pt 16), 3677-3689. doi: 10.1113/jphysiol.2012.230268
